# *QuickStats:* Age-Adjusted Rates* of Firearm-Related Homicide,^†^ by Race,^§^ Hispanic Origin, and Sex — National Vital Statistics System, United States, 2021

**DOI:** 10.15585/mmwr.mm7226a9

**Published:** 2023-06-30

**Figure:**
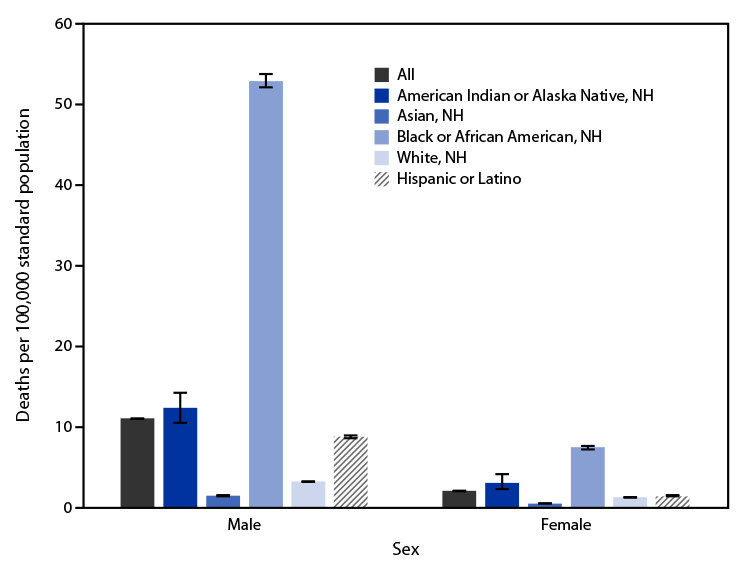
In 2021, among males, Black or African American (Black) males had the highest age-adjusted rate of firearm-related homicide (52.9 deaths per 100,000 standard population), and Asian males had the lowest rate (1.5). Among females, Black females had the highest rate (7.5), and Asian females had the lowest rate (0.5). Males had higher rates than females across all race and Hispanic origin groups.

**Source:** National Center for Health Statistics, National Vital Statistics System, Mortality Data, 2021. https://www.cdc.gov/nchs/nvss/deaths.htm

For more information on this topic, CDC recommends the following link: https://www.cdc.gov/vitalsigns/firearm-deaths/index.html

